# Climate warming drives population trajectories of freshwater fish

**DOI:** 10.1073/pnas.2410355121

**Published:** 2024-12-09

**Authors:** Timothy M. Brown, Joseph O’Connor, Martin J. Genner

**Affiliations:** ^a^School of Biological Sciences, University of Bristol, Bristol BS8 1TQ, United Kingdom; ^b^School of Biology, Faculty of Biological Sciences, University of Leeds, Leeds LS2 9JT, United Kingdom

**Keywords:** global heating, riverine, abundance, timeseries, extinction

## Abstract

Climate change has emerged as a key threat to biodiversity, leading to broad-scale shifts in distributions of marine and terrestrial species as they attempt to track thermally suitable habitat. By contrast, our understanding of climate responses of freshwater species is relatively undeveloped, limiting our knowledge of whether projected warming will lead to freshwater biodiversity loss. Here, we linked a multicontinental database of riverine fish population abundance time series collected from 1958 to 2019 to temperature data from the same period. Across the sampled localities, waters warmed by 0.21 °C per decade (annual maximum of monthly temperatures). We tested whether fish responded to this change by i) increasing abundance at the cooler poleward limit of species distributions—predicted if warming has opened new opportunities—and ii) decreasing abundance toward the equatorward limit of distributions—predicted if temperatures have exceeded tolerance thresholds. We found that observed population trends were consistent with both of these expected patterns from climatic warming and that the trends were more pronounced in time series covering the longer time periods of 30+ y. The responses consistent with climate change were most evident in species with larger body sizes, higher trophic levels, river–sea migratory behavior, and more widespread distributions. Moreover, positive abundance responses to warming were more likely at higher altitudes where conditions tend to be cooler. These findings indicate that projected future warming will likely lead to widespread shifts in riverine community structure, including abundance declines at the trailing edge of species distributions.

## Significance

Understanding how the population abundance of freshwater species has responded climatic warming over recent decades is important for predicting how they are likely to be impacted by future conditions. By coupling a multicontinental database of freshwater fish abundance with climate data between 1958 and 2019, we show that recent increases in stream temperatures are associated with increases in fish populations at the poleward limit of species’ ranges and decreases in abundance toward their equatorward limit. This pattern was consistent across geographical regions and taxonomic groups but was also mediated by altitude and species traits. These findings suggest that climate change has had a definable impact on fish population dynamics, highlighting the importance of conservation management that integrates climate risk.

One commonly reported response of species to changing climate is shifts in their distributional ranges as they track suitable bioclimatic conditions (1, 2). In response to warming temperatures, species are expected to track optimal thermal conditions by moving to higher latitudes and higher elevations where temperatures are currently cooler (3). In practice, range shifts of species are highly complex and variable (4), being dependent on species sensitivity to climate and adaptive capacity, as well as spatial heterogeneity in climate exposure, spatial barriers to species movement, and interspecies interactions (5). There is particular concern about the threat of species extinctions at the trailing edge of their distributions (6), with risks elevating if species are unable to adapt or move rapidly enough to tolerate new climate conditions, such as when intrinsic factors or extrinsic barriers prevent dispersal to more favorable habitat (7).

Air temperature is a key determinant of freshwater surface water temperatures (8–10), and it is projected that increases of 2 °C in global air temperatures will lead to increases of 1.3 °C in freshwater surfaces (11). These projected increases will have profound impacts on the physical and chemical properties of freshwater ecosystems, and the biology of the organisms that inhabit them (12, 13). As ectothermic organisms, freshwater fish are particularly vulnerable to increases in water temperatures (14), being affected from the cellular to community level (15), and across all stages of their life history from embryonic development to spawning (16), including key processes such as recruitment and mortality (17, 18). The metabolic rate of fishes increases with temperature (19), leading to increased scope for growth, swimming speed, and activity up to an optimal point. However, increased metabolic rate comes with increased metabolic cost, and at high temperatures, the food required to sustain metabolic processes may exceed food availability, leading to declining metabolic performance and survivorship (20). Moreover, at higher water temperatures, surface water holds less dissolved oxygen (21), decreasing the supply available for respiration and leading to hypoxia, reduced growth, and mortality (22).

Many studies have modeled the nature and extent of range shifts to assess the vulnerability of riverine fish species to climate change. In such studies, often the magnitude and duration of warm water temperatures have been projected to exceed those currently experienced, leading to declines in the availability of thermally suitable habitat (23–26). However, there is also evidence that some warm-water-adapted species may benefit locally from warming (27, 28). Some studies have additionally considered the ability of species to respond to new thermal regimes through evolutionary adaptation or dispersal. These models have produced similar results to those anticipated by correlative models, forecasting net losses of thermally suitable habitat for freshwater fish, as thermal regimes are likely to shift faster than species can disperse (29) or evolve (30). These studies also point to how species-specific responses to warming are likely to be highly idiosyncratic, as species characteristics, such as body size, can strongly mediate species responses to climate change by impacting both sensitivity and resilience (31).

Observational evidence has an important role in providing validation of biological models, as well as informing conservation managers of the urgency and type of conservation action necessary to curb future biodiversity loss (32). Spatially and temporally extensive studies are particularly important as they allow us to identify large-scale patterns of ecological change that can be robustly attributed to climate change (33). For marine fishes, there is compelling evidence of distributional shifts to higher latitudes (1) and local abundance changes reflecting thermal conditions (34, 35), leading to increased dominance of warm-water fishes in fisheries catches (36). In comparison, evidence of climate change impacting freshwater species is much less prominent, potentially because freshwater fish populations have been strongly influenced by numerous other stressors including habitat degradation, land-use change, and pollution (37). Responses to climate change may also be influenced by the lack of connectivity between freshwater systems, that limits active movement and dispersal to thermally suitable habitat, both horizontally and vertically (38). Nevertheless, recent work has shown an increasing dominance of warm water species at the community level in global riverine fish assemblages (39), supporting taxonomically and geographically focused studies of climate impacts (40–45). Collectively, these studies are generally consistent with some freshwater fish species undergoing abundance increases at higher latitudes and elevations. Here, we contribute to this evidence with a multicontinental test of a key prediction of the impact of climate change on freshwater fish—that riverine fish populations toward the poleward (cooler) limit of a species’ range will respond positively to climate warming, whereas populations toward the equatorward (warmer) limit of a species’ range will experience unfavorable temperatures leading to population declines. We also test how the duration of a time series affects the resolution of this latitudinally dichotomous response to warming, and whether responses are mediated by life history traits, the distributional ranges of species, and the altitude of survey locations.

## Results

### Survey Data and Warming Freshwaters.

**Fig. 1.**
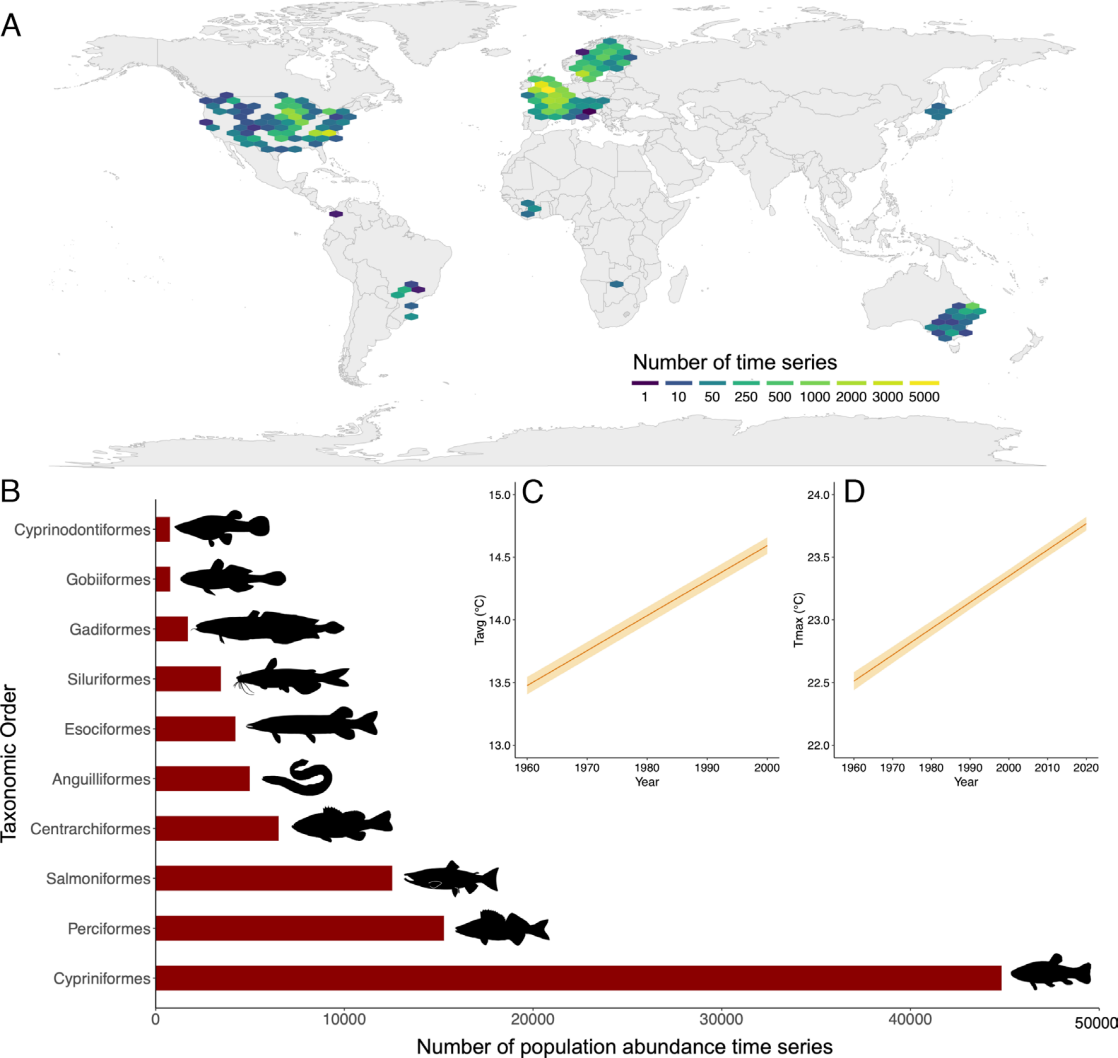
(*A*) The global distribution of the time series analyzed, (*B*) The taxonomic diversity of the population abundance time-series data analyzed; (*C*) change in average of the monthly maximum temperatures in a year (T_avg_) and (*D*) change in maximum of the monthly maximum temperatures in a year (T_max_), for sampled localities across the time period. Shaded areas indicate 95% CI. Images are from PhyloPic (https://www.phylopic.org/). The *Lota lota* image is by E. Edmonson and T.J. Bartley (https://creativecommons.org/licenses/by-sa/3.0/).

We used RivFishTime, a database of freshwater fish population abundance time series (46). In total, we analyzed 10,220 time series of riverine community catch data, including 91,935 population-specific abundance measurements from 632 species, from 9,989 locations sampled worldwide. The analyzed data spanned the period 1958 to 2019, and each time series had a minimum of two survey years and covered a minimum time span of 10 y (*SI Appendix*, Fig. S1). The analyzed dataset was dominated geographically by data from Europe, the Eastern USA, and South-eastern Australia (Fig. 1*A*), and taxonomically by Cypriniformes, Perciformes, and Salmoniformes (Fig. 1*B*). To match each of population-specific abundance measurements in the fish time series, we obtained estimates of two water temperature variables; the average of the monthly maximum temperatures in a year (herein T_avg_; Fig. 1*C*) and the maximum of the monthly maximum temperatures in a year (herein T_max;_ Fig. 1*D*). Linear mixed models showed a significant warming trend across our survey locations (Dataset S01) in both temperature variables (T_avg_, 0.279 ± SE 0.003 °C per decade; T_max_, 0.210 ± SE 0.006 °C per decade).

### Population Responses Depend Upon Their Location in the Species Latitudinal Range.

**Fig. 2.**
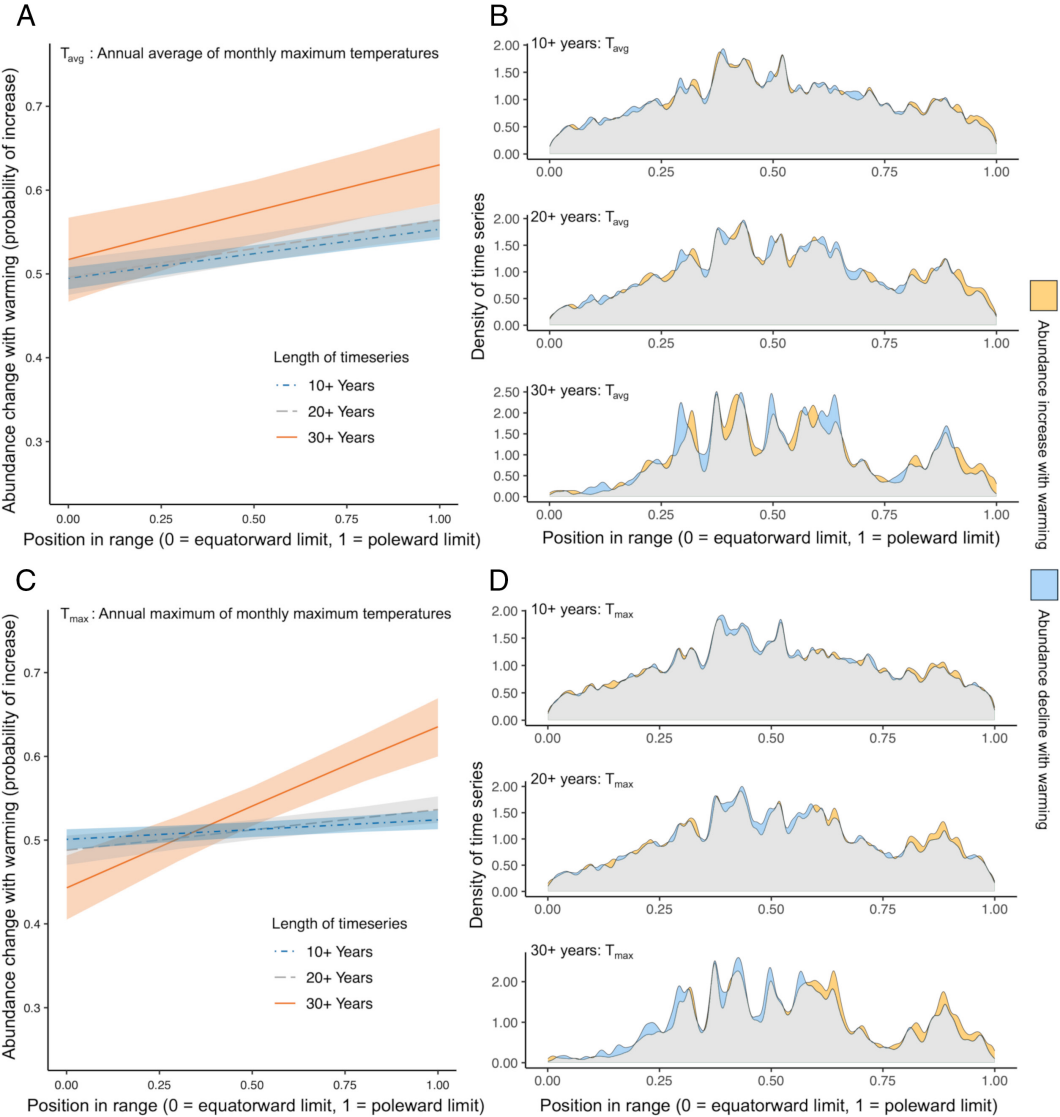
Abundance changes over the study period in response to increases in (*A* and *B*) the annual average of monthly maximum temperature (T_avg_), and (*C* and *D*) the annual maximum of monthly maximum temperatures (T_max_), using time series of different durations. (*A* and *C*) Modeled predicted probability of abundance change in response to warming temperature from linear mixed models, with shaded areas indicating 95% CI. (*B* and *D*) Density plots indicating the latitudinal distributions of populations showing an overall positive and negative response to warming during the time series. Gray shading shows an equal number of time series undergoing abundance increases or declines in response to warming; green or black shading indicates where abundance increases or declines in response to warming dominate, respectively.

We obtained information on the latitudinal distributions of species from the Global Biodiversity Information Facility (GBIF) database (47), enabling us to test whether the relative location of a sampled population within the latitudinal range of the species predicted the population response to temperature. For each time series in our dataset, we calculated the slope of the linear regression between population abundance and temperature, assigning a binary value of 1 or 0 to reflect a population increase with increasing temperature or population decrease with increasing temperature respectively. To quantify the relationship between population response and position of the population in the species’ range we used generalized linear mixed models. Our results clearly supported the hypothesis that population-level temperature responses were predicted by the relative position of the population in the species range (Table 1 and Dataset S02). Populations were more likely to show abundance increases in response to warming temperatures toward the poleward limits of their range, while populations were more likely to decline with warming temperatures when they are closer to the equatorward limit of the species. The magnitude of the dichotomous effect of latitudinal position on abundance change depended on the duration of the time series used in the model, with the longer time series showing more pronounced effects (Fig. 2*A* and *C*, Table 1 and Dataset S02). The effect was greatest when models used the longest time series (30+ y) and the annual maximum of monthly maximum temperatures (T_max_), rather than the annual average of monthly maximum temperatures (T_avg_). Consistent with predictions, population declines were most prominent in the most equatorward sector of species ranges, and population increases were most prominent in the most poleward sector of species ranges (Fig. 2 *B* and *D*).

**Table 1.** The effect of relative position of a population in a species range on the probability of a population increase in response to increasing water temperature

Temperature variable	Time-series duration (years)	Estimate effect of position in range	SE of estimate	z	*P*
T_avg_	10+	0.2350	0.0310	7.572	<0.001
T_avg_	20+	0.2740	0.0499	5.493	<0.001
T_avg_	30+	0.4648	0.1269	3.662	<0.001
T_max_	10+	0.0935	0.0306	3.056	0.002
T_max_	20+	0.1944	0.0483	4.029	<0.001
T_max_	30+	0.7847	0.1225	6.403	<0.001

T_avg_ is the average of monthly maximum temperatures in a survey year. T_max_ is the maximum of monthly maximum temperatures in a survey year.

### Geography and Species Traits Mediate Population Responses to Warming.

**Fig. 3.**
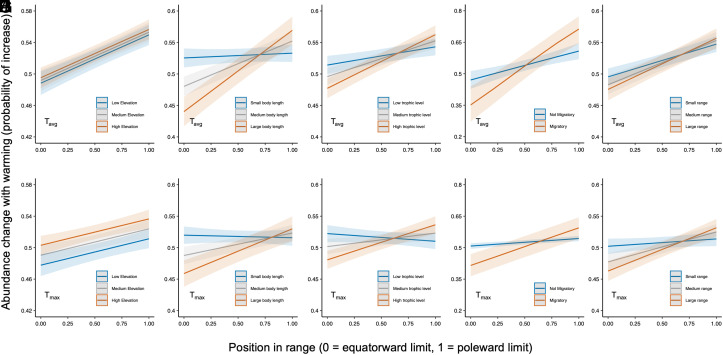
Effect of elevation, body length, trophic level, river–sea migratory behavior, and geographic range on the population responses of freshwater fish populations to increasing stream temperature. (*A*–*E*) responses to T_avg_ – the average of monthly maximum temperatures in a survey year – for time series 10+ y in duration. (*F*–*J*) responses to T_max_ – the maximum of monthly maximum temperatures in a survey year – for time series 10+ y in duration. The three lines show the relationship between position in range and abundance change for the median value of the secondary variable and one quartile either side. Shaded areas represent 95% CI.

Since higher altitudes experience cooler temperatures, we tested the prediction that populations at the highest elevations are more likely to exhibit population increases as water temperatures increase. Our results confirmed that populations that were sampled at higher altitude were significantly more likely to show an overall increase in abundance over the study periods, relative to those at lower latitudes (Fig. 3 *A* and *F*, Table 2 and Dataset S03). We next investigated how species traits determine the probability of dichotomous abundance change responses to warming temperatures across the latitudinal range of species. We found that the likelihood of a dichotomous response was significantly dependent on both the maximum length, trophic level, and the river–sea migratory behavior of a species (Table 2 and Datasets S04–S06). Specifically, larger-bodied species, species from higher trophic levels, and species with river–sea migratory behavior were all more likely to undertake abundance increases at the poleward edge of species ranges, and abundance declines at the equatorward edge of species ranges (Fig. 3 *B*–*D* and *G*–*I*). Next, we tested whether the overall latitudinal range size of a species influenced the probability of a dichotomous response to warming across their latitudinal range. Our results demonstrated that species with larger latitudinal ranges are significantly more likely to exhibit population increases nearing the poleward limits of their range as temperatures increase, and population declines nearing the equatorward limit of their range (Fig. 3 *E* and *J*, Table 2 and Dataset S07).We found no clear evidence of differences between populations sampled in the northern hemisphere and those sampled in the southern hemisphere in abundance change responses to warming temperatures across the latitudinal range of species (Dataset S08).

**Table 2.** The effect of the elevation of sampling location (position in range) on population responses of freshwater fish to increasing water temperature (T_avg_ and T_max_)

Variable	Temperature variable	Effect estimate	Interaction effect estimate	Estimate SE	z	*P*
Elevation	T_avg_	0.0138	–	0.0080	1.736	0.0826
Elevation	T_max_	0.0506	–	0.0078	6.443	<0.001
Total length	T_avg_	–	0.2532	0.0319	7.943	<0.001
Total length	T_max_	–	0.1561	0.0312	5.010	<0.001
Trophic level	T_avg_	–	0.2000	0.0312	6.411	<0.001
Trophic level	T_max_	–	0.2401	0.0307	7.833	<0.001
Migratory behavior	T_avg_	–	0.3018	0.0847	3.561	<0.001
Migratory behavior	T_max_	–	0.5805	0.0856	6.781	<0.001
Range size	T_avg_	–	0.1173	0.0293	4.007	<0.001
Range size	T_max_	–	0.2289	0.0284	8.050	<0.001

The interaction between species traits (body size, trophic level, river–sea migratory behavior, and latitudinal range size) and sampling location on population responses to increasing water temperature (T_avg_ and T_max_). Results are shown for time series 10+ y in duration. – indicates parameter not estimated in the model.

## Discussion

Our analyses demonstrate that freshwater fish populations have shown a widespread and consistent pattern of population change since the 1950 s, exhibiting abundance increases with increasing temperatures in poleward populations and abundance declines with increasing temperatures in equatorward populations. This corresponds with expectations that for freshwater fish species with unimodal thermal response curves (25), climate warming will lead to an amelioration of excessively cool conditions at the colder (poleward) limit of distributions encouraging population growth, and will lead to temperatures exceeding thermal tolerances of species at the warmer (equatorward) limit of distributions driving population declines. The “sign-switching” dichotomous pattern of abundance change at different range limits matches that observed in a broad range of taxa (34) and can been described as a fingerprint of anthropogenic climate change. This is based on the hypothesis that only responses to thermal environmental change can consistently account for the opposite trends at opposite latitudes within the same species, unlike more spatially heterogenous drivers of biotic change such as habitat modification, pollution, and shifts in land use (37).

The thermal change across sampling sites in the analyzed dataset was 0.21 °C per decade, which broadly corresponds with warming observed from in situ water temperature measurements (48, 49). It is plausible that this magnitude of warming may have longer-term impact on performance, including growth rates, reproductive success, locomotion, and behavior (49, 50). Additionally, there is potential for warming to lead to indirect effects on populations, through changes to the extent and timing of food resource availability, and the prevalence of disease (49). We may predict such effects to be most apparent in longer-term datasets, and in line with this expectation, we found that the effect size of sampling location on abundance change increased with the duration of the time series used in the model. This pattern is consistent with short-term variation associated with interannual changes in stream temperature masking long-term climatic trends (51). This result has significance for the study of long-term population dynamics of freshwater fish as it suggests that the longer fish populations are exposed to warming, the more likely they are to exhibit a quantifiable abundance response.

We found that the effect of sampling location on abundance change was larger in the models that used T_max_ rather than T_avg_, suggesting that thermal extremes may have a key role in determining abundance changes. Similar results have been reported in a meta-analysis of local extinctions (i.e., population decreases) of plant and animal species caused by climate change, where more extinctions were found in locations with the greatest increase in yearly temperature extremes (52). This supports the notion that the thermal stress that organisms experience is a result of the magnitude of thermal change, as well as the duration of exposure to these extreme temperatures (16). Collectively these results are indicative of the impact that extreme acute events can have on population abundance (53).

Our analysis showed that the impacts of climate change are not experienced evenly across the latitudinal range of a species. We typically observed that the magnitude of increase in the likelihood of a positive abundance change at the poleward side of a species range was larger than the magnitude of decrease in the likelihood of a positive change at the equatorward side. This is potentially due to sampling bias associated with biological abundance surveys, whereby populations at the trailing edge of a species distribution are likely to be small and isolated which leads to undersampling (32). The uneven trend may also reflect a lag in the response of population abundance to warming temperatures; populations can persist in locations where species’ thermal tolerances are exceeded through phenotypic plasticity (10) or due to the existence of sink habitats where populations are sustained by the dispersal of individuals from more habitable localities (54). Over time this can lead to the accumulation of an extinction debt in freshwater fish populations that will be repaid due to continued exposure to high temperatures (55). Lagged responses to climate change have been observed in the fish communities of French streams (43), and such delayed responses may also explain our observations that longer time series are associated with a stronger latitudinally dependent abundance change effect.

We found that the altitude at which a population was sampled influenced how the population responded to increasing water temperatures, with species sampled at higher elevations more likely to show population increases. This matches our expectations given that streams at higher altitudes experience cooler thermal regimes and are subsequently more likely to be exposed to more favorable temperatures with warming. Our finding is in accordance with studies that have tracked altitudinal range shifts for freshwater fish species, which have shown that ranges have shifted upslope as stream temperatures have warmed (43, 44). This result draws attention to the potential for high-altitude streams or headwaters to act as thermal refugia, emphasizing the need to safeguard and restore freshwater connectivity to allow species to access these habitats (56). Set against this, streams tend to decline in size and habitat heterogeneity with altitude (57), potentially limiting their ability to provide thermal refugia to all species due to crowding effects (58). Moreover, headwaters can function as sky islands (14), imposing strong limits on the ability of fish to respond to further climate change (59). It is also possible that high-altitude water sources, for example, glacier meltwater, may partially buffer river systems against warming and therefore mediate population trajectories.

The dispersal ability of freshwater fish is intrinsically limited due to the lack of freshwater connectivity between catchments. Therefore the responses we observe are most likely to be linked to movement within catchments to more favorable environments, or due to local population abundance change in the absence of any movement. Importantly, however, we also demonstrated considerable variation among individual species mediated by species traits. We observed that species with larger maximum body length, species of a higher trophic level, and species with river–sea migratory behavior, were more likely to undergo abundance increases at the poleward side of their ranges, and abundance declines at the equatorward side of their ranges. These species traits were positively correlated with one another across the species analyzed, consistent with findings of studies that have linked larger body sizes to migratory behavior (60, 61). These patterns open up the possibility that responses to warming we observed are in part mediated by dispersal ability, with dispersion through the marine environment allowing species to take advantage of newly available habitat, or escape from disappearing thermal habitat. In this study, we have not subdivided the populations of river–sea migratory species into those that are fully migratory, those that are partially migratory (62), and those that are landlocked. Future work exploring time-series data using this approach would help to clarify how migratory behavior mediates responses of the fish species climate change.

We observed that species of higher trophic level were more likely to undertake abundance increases at the poleward side of their ranges, and abundance declines at the equatorward side of their ranges. This pattern is consistent with other work showing that trophic level is a good predictor of population expansions among freshwater fish populations at the cold limit of their ranges (31). Plausibly, predatory fish species at higher latitudes may be able to take advantage of newly available food resources resulting from the productivity increases that accompany warming temperatures (20). Furthermore, declines in predatory species at the warm side of their ranges may be due to the increase in metabolic rates as water temperatures rise, in combination with the negative effect of water temperatures on predatory behavior (15). Species at higher trophic levels have larger individual energy demands, hence, they may be more vulnerable to insufficient food availability and increased thermal stress. Consideration must also be given to the interactions between species across different trophic levels, as the physiological depression of predators at higher temperatures may reduce predation pressure, allowing increased abundance of fish species belonging to lower trophic levels toward the equator (5, 42). The extent to which we can estimate the importance of such species interactions from our analyses is limited, but the significance of the interaction between trophic level and sampling location supports previous assertions that shifting predator–prey interactions will be an important proximate cause of species extinctions as a result of climate change (63).

Finally, we showed that as temperatures have warmed, species with larger range sizes were more likely to exhibit abundance increases near the poleward limits of their ranges and more likely to exhibit abundance decreases nearer the equatorward limits of their ranges. We suggest that our results derive from species with larger ranges providing a greater ability to observe climatic influences, largely due to the enhanced range of conditions that widely distributed species will be exposed to. Species with large range size are also potentially able to tolerate a wider variety of environmental and habitat conditions, which should allow them to take full advantage of increases in the availability of thermally suitable habitat through population increases (64).

In summary, this study provides a clear demonstration of a global pattern of sign-switching population change across the distributions of freshwater fish species. We have shown that rising water temperatures during climate warming have left an indelible fingerprint of increases in freshwater fish populations toward the poleward limits of their distributions, and population declines toward their equatorward limits. Despite the large geographical range and taxonomical breadth of the data used in this study, there are significant regional gaps in the analysed dataset, particularly over the Afrotropical and Neotropical realms (46). These gaps are important to recognize given that possible vulnerability of species in these areas to climate change. There is also potential for the climate responses of species in tropical habitat to differ from those species in colder regions (28, 57). Moreover, this study highlights the need to assess the rates at which freshwater fish species are colonizing new habitat at the leading edge of their historical distributions, in part to compensate for the declines we have identified at the trailing edge of their distributions. Particular focus must be given to how the unique structure and connectivity of riverine systems creates limitations on the capability of freshwater fish species to track thermal regimes (65), and whether trait-based vulnerability assessments can be used alongside conventional estimates of climate exposure when planning conservation measures (66). This study draws attention to the extent to which recent climate change has already impacted freshwater ecosystems, underlining the need for the implementation of climate-targeted conservation interventions that aim to enhance the capacity of species to withstand future climate change (67).

## Materials and Methods

### Freshwater Fish Abundance Data.

The freshwater fish population abundance data were obtained from the RivFishTime database (46). This database comprises time series of community data of riverine fishes from regional and national monitoring schemes and individual academic surveys. For inclusion in our analyses, time series required a known and consistent sampling location and methodology, and only those from lotic systems were included. Abundance data were primarily in the format of individual counts, catch per unit effort, or individuals per 100 m^2^. For time series in which multiple observations were taken during a single year, we calculated the mean average value of these observations. Species that were sampled at least once in time series of community catch data were assumed to have an abundance of zero for other sampling events in that time series if no abundance value was present. Due to the incomplete temporal coverage of the RivFishTime data by the TerraClimate database (used for obtaining matching temperature data), any abundance records that were collected earlier than 1958 were excluded from the study. Included species had corresponding species trait data available (see below). After filtering, the analyzed data included 10,220 time series of community catch data, encompassing 91,935 species-specific abundance records with accompanying metadata on geographical location, across 632 species, and 9,995 locations worldwide. The analyzed records span the period 1958 to 2019 with each survey composed of a minimum of 2 y surveys and a minimum time span of 10 y.

### Water Temperature Data.

We estimated water temperature from air temperature data from the TerraClimate dataset, which provides high spatial and temporal resolution climate data for global terrestrial surfaces from 1958 to 2020 (68). The TerraClimate database was queried using the exact latitude, longitude, and year for each RivFishTime abundance record, using the R package climateR v.0.3.5 (69) and the “getTerraclim” function. TerraClimate provides high-spatial resolution (1/24°, ~4 km) monthly climate data. For each abundance record, T_avg_ (the annual average of monthly maximum temperatures), and T_max_ (the annual average of monthly maximum temperatures) were extracted. From these TerraClimate air temperature data, we derived water temperature using a global nonlinear regression model with the following equation (70, 71):

$$T_{\mathrm{water}}=\frac{32}{\left[1+e^{\left(-0.13T_{\mathrm{air}}+1.94\right)}\right]}.$$

T_water_ and T_air_ refer to water temperature and air temperature respectively. Data were transformed from air to water temperatures prior to calculating the mean annual temperature and maximum mean monthly temperature. This global nonlinear regression model has been previously validated for several time series reflecting different climatic regimes (71) and used in a previous macroscale analysis of fish responses to stream temperature change (30).

### Species Distribution Data.

We estimated species ranges using data from the GBIF (47). Point occurrence data were downloaded from GBIF using the R package rgbif v.3.7.9 (72), with the “occ_search” function, using the species names provided in the RivFishTime database. The most extreme equatorward and poleward observations (defined as 1% at each tail) were removed to account for incorrectly entered occurrences or identification errors, and the maximum and minimum latitude of occurrence for each species was recorded. For each time series, the latitude of the sampling location of the population relative to the entire species latitudinal range was calculated using the equation:

$$\mathrm{Position}\;\mathrm{in}\;\mathrm{Species}\;\mathrm{Range}\;=\frac{\left(\mathrm{Observed}\;\mathrm{Latitude}\;-\;\mathrm{Equatorward}\;\mathrm{Limit}\right)}{\left(\mathrm{Poleward}\;\mathrm{Limit}\;-\;\mathrm{Equatorward}\;\mathrm{Limit}\right)}.$$

If a species range spanned the equator and the range included a tropical distribution, we treated the distribution as having a southern and northern component, treating the equator as the equatorward limit. Some temperate species with a native northern hemisphere had been introduced to southern hemisphere, and for those species, we only studied the distribution in the native northern hemisphere range.

### Altitude Data.

We used the R package elevatR v.0.99.0 (73), using the “get_elev_point” function, which allows the user to extract point elevation data of bare-earth terrain heights (given in meters above sea level) for given coordinates, from the Amazon Web Services terrain tiles.

### Species Trait Data.

Taxonomic and trait data were obtained from Fishbase (74) using the R package rfishbase v.4.1.2 (75). We focused analyses on maximum length (total length, mm) and trophic level. Trophic level of a consumer fish is defined as 1 + mean trophic level of the food items. The trophic level of food items is defined as 1 = primary producer, 2 = primary consumer, Level 3 = secondary consumer, and Level 4 = tertiary consumers. Level 5 represents an apex predator. If there were no data on the length or trophic level of a species, the species was excluded from the study. Species were classified as migratory if they are known to be anadromous, catadromous, or amphidromous, following Fishbase (74).

### Temperature Variation at Sampled Locations.

To assess how stream temperatures at our sampled localities changed across the study period, we ran linear mixed models to measure the effect of time on water temperature with location treated as a random effect. The models were fitted using the “lmer” function in the R package lme4 v.1.1-35.3 (76), with the formula: water temperature ~ year + (1 | location). Coefficients from models were plotted using the “effect” function from R package effects v.4.2-2 (77, 78).

### Modeling Abundance Responses to Temperature.

For each population abundance time series in our dataset, we calculated the direction of abundance change in relation to increasing temperature using the slope of the regression between standardized sampled abundance (standardized for the species and time series, so that the mean = 0, SE = 1) and temperature (for both T_avg_ and T_max_). Each time series was assigned a binary value of 1 or 0, corresponding to a positive or negative abundance change in response to increasing temperature respectively. We focused our analyses on binary responses, although key hypotheses were also repeated with using slopes of the association between standardized sampled abundance and temperature.

To test the hypothesis that climate change has driven poleward increases and equatorward declines in freshwater fish populations, we used binomial generalized linear mixed models using a logit link function, employing the “sampling location” (relative latitudinal position of a sampled population in a species range) as a predictor variable, species as a random factor, and abundance changes during warming as the response variable. These models were fitted to both T_avg_ and T_max_ using the glmer function in the R package lme4 v.1.1-35.3 (76) with the formula: abundance change ~ sampling location + (1 | species), family = binomial (“logit”). To investigate how the duration of the time series used in the model affected the relationship between sampling location and abundance change, we applied the same modeling approach to subsets of the data. Specifically, our analyses with 10+ y of data contained all analyzed time series, our analyses with 20+ y of data contained a smaller subset of time series of duration 20 y or more, and our analyses with 30+ y of data contained an even smaller subset of time series of duration 30 y or more. Coefficients from models were plotted using the effect function from R package effects v.4.2-2 (77, 78).

To determine how elevation influenced the effect of temperature change on freshwater fish populations, we used elevation alongside sampling position in our generalized linear mixed models with the formula: abundance change ~ Sampling location + elevation + (1 |species), family = binomial (link = “logit”). To determine how species traits [maximum length (total length cm), trophic level, range size (km)] influenced the effect of temperature change on freshwater fish populations, we tested for an interaction between the trait and sampling location in our generalized linear mixed models with the formula: abundance change ~ Sampling location * variable + (1 | species), family = binomial (link = logit). The same approach was used to test whether patterns of abundance change relative to sampling location were consistent between the northern and southern hemispheres. Elevation and species trait data were scaled prior to analysis, using the scale function in the R package standardize v.0.2.2 (79). Models were constructed for both T_avg_ and T_max_ data using the “glmer” function in the R package lme4 v.1.1-35.3 (76). Effects of variables were visualized using the “plot.model” function from the R package sjPlot v.2.8.15 (80). Maximum length, trophic level, range size were positively correlated with one another (Dataset S09), but were analyzed independently of one another in this study.

To test whether the resolved patterns in binomial response (i.e., abundance increases or declines) were also seen in continuous response data, we used the slope of the regression between standardized sampled abundance and temperature as a response variable in linear mixed models; the two methods gave comparable results (Dataset S10). All analyses were conducted in R v.4.4.0 (81), with figures generated using ggplot2 v.3.5.1 (82) and statistical tests of linear models undertaken with lmerTest v.3.1-3 (83).

## Supplementary Material

Appendix 01 (PDF)

Dataset S01 (XLSX)

Dataset S02 (XLSX)

Dataset S03 (XLSX)

Dataset S04 (XLSX)

Dataset S05 (XLSX)

Dataset S06 (XLSX)

Dataset S07 (XLSX)

Dataset S08 (XLSX)

Dataset S09 (XLSX)

Dataset S10 (XLSX)

## Data Availability

Abundance data were from the RivFishTime database (46) available through the iDiv Biodiversity Portal (https://doi.org/10.25829/idiv.1873-10-4000). Species range data were sourced from the GBIF (https://doi.org/10.15468/dl.37y38n). Climate data were sourced from Terraclimate (68). Species trait data were sourced from FishBase (74). Analysis code and analyzed data are available via GitHub (https://doi.org/10.5281/zenodo.14037297; https://github.com/genner-lab/freshwater_fish_climate).

## References

[1] A. L. Perry, P. J. Low, J. R. Ellis, J. D. Reynolds, Climate change and distribution shifts in marine fishes. Science **308**, 1912–1915 (2005). 15890845 10.1126/science.1111322

[2] C. Parmesan, G. Yohe, A globally coherent fingerprint of climate change impacts across natural systems. Nature **421**, 37–42 (2003). 12511946 10.1038/nature01286

[3] I.-C. Chen, J. K. Hill, R. Ohlemüller, D. B. Roy, C. D. Thomas, Rapid range shifts of species associated with high levels of climate warming. Science **333**, 1024–1026 (2011). 21852500 10.1126/science.1206432

[4] F. A. La Sorte, W. Jetz, Tracking of climatic niche boundaries under recent climate change. J. Anim. Ecol. **81**, 914–925 (2012). 22372840 10.1111/j.1365-2656.2012.01958.x

[5] D. Kishi, M. Murakami, S. Nakano, K. Maekawa, Water temperature determines strength of top-down control in a stream food web. Freshw. Biol. **50**, 1315–1322 (2005).

[6] J. J. Wiens, Climate-related local extinctions are already widespread among plant and animal species. PLoS Biol. **14**, e2001104 (2016). 27930674 10.1371/journal.pbio.2001104 PMC5147797

[7] M. C. Urban, Accelerating extinction risk from climate change. Science **348**, 571–573 (2015). 25931559 10.1126/science.aaa4984

[8] S. S. Kaushal , Rising stream and river temperatures in the United States. Front. Ecol. Environ. **8**, 461–466 (2010).

[9] D. Markovic, U. Scharfenberger, S. Schmutz, F. Pletterbauer, C. Wolter, Variability and alterations of water temperatures across the Elbe and Danube river basins. Clim. Change **119**, 375–389 (2013).

[10] J. H. Knouft, D. L. Ficklin, The potential impacts of climate change on biodiversity in flowing freshwater systems. Annu. Rev. Ecol. Evol. Syst. **48**, 111–133 (2017).

[11] M. T. H. van Vliet, F. Ludwig, J. J. G. Zwolsman, G. P. Weedon, P. Kabat, Global river temperatures and sensitivity to atmospheric warming and changes in river flow. Water Resour. Res. **47**, W02544 (2011).

[12] A. J. Reid , Emerging threats and persistent conservation challenges for freshwater biodiversity. Biol. Rev. **94**, 849–873 (2019). 30467930 10.1111/brv.12480

[13] B. R. Scheffers , The broad footprint of climate change from genes to biomes to people. Science **354**, aaf7671 (2016). 27846577 10.1126/science.aaf7671

[14] F. Pletterbauer, A. Melcher, W. Graf, “Climate change impacts in riverine ecosystems” in Riverine Ecosystem Management: Science for Governing Towards a Sustainable Future, Aquatic Ecology Series, S. Schmutz, J. Sendzimir, Eds. (Springer, 2018), pp. 203–223

[15] C. T. Graham, C. Harrod, Implications of climate change for the fishes of the British Isles. J. Fish Biol. **74**, 1143–1205 (2009). 20735625 10.1111/j.1095-8649.2009.02180.x

[16] B. Jonsson, N. Jonsson, A review of the likely effects of climate change on anadromous Atlantic salmon *Salmo salar* and brown trout *Salmo trutta*, with particular reference to water temperature and flow. J. Fish Biol. **75**, 2381–2447 (2009). 20738500 10.1111/j.1095-8649.2009.02380.x

[17] A. D. Nunn, P. A. Frear, M. Lee, I. G. Cowx, Is there evidence for a shift in fish growth and recruitment success linked to climate change? J. Fish Biol. **77**, 1780–1792 (2010). 21078090 10.1111/j.1095-8649.2010.02751.x

[18] R. D. Bassar, B. H. Letcher, K. H. Nislow, A. R. Whiteley, Changes in seasonal climate outpace compensatory density-dependence in eastern brook trout. Glob. Chang. Biol. **22**, 577–593 (2016). 26490737 10.1111/gcb.13135

[19] K. M. Jeffries , Effects of high temperatures on threatened estuarine fishes during periods of extreme drought. J. Exp. Biol. **219**, 1705–1716 (2016). 27252456 10.1242/jeb.134528

[20] J. D. Reist , An overview of effects of climate change on selected Arctic freshwater and anadromous fishes. Ambio **35**, 381–387 (2006). 17256642 10.1579/0044-7447(2006)35[381:aooeoc]2.0.co;2

[21] S. R. Carpenter, E. H. Stanley, M. J. Vander Zanden, State of the World’s freshwater ecosystems: Physical, chemical, and biological changes. Annu. Rev. Environ. Resour. **36**, 75–99 (2011).

[22] N. E. Jones, I. C. Petreman, Relating extremes of flow and air temperature to stream fish communities. Ecohydrology **6**, 826–835 (2013).

[23] M. T. H. van Vliet, F. Ludwig, P. Kabat, Global streamflow and thermal habitats of freshwater fishes under climate change. Clim. Change **121**, 739–754 (2013).

[24] V. Barbarossa , Threats of global warming to the world’s freshwater fishes. Nat. Commun. **12**, 1701 (2021). 33723261 10.1038/s41467-021-21655-w PMC7960982

[25] O. Kärcher, D. Hering, K. Frank, D. Markovic, Freshwater species distributions along thermal gradients. Ecol. Evol. **9**, 111–124 (2019). 30680100 10.1002/ece3.4659 PMC6342105

[26] D. Markovic, S. F. Carrizo, O. Kärcher, A. Walz, J. N. W. David, Vulnerability of European freshwater catchments to climate change. Glob. Chang. Biol. **23**, 3567–3580 (2017). 28186382 10.1111/gcb.13657

[27] L. Buisson, W. Thuiller, S. Lek, P. Lim, G. Grenouillet, Climate change hastens the turnover of stream fish assemblages. Glob. Chang. Biol. **14**, 2232–2248 (2008).

[28] T. Oberdorff , Opinion Paper: How vulnerable are Amazonian freshwater fishes to ongoing climate change? J. Appl. Ichthyol. **31**, 4–9 (2015).

[29] J. Radinger , The future distribution of river fish: The complex interplay of climate and land use changes, species dispersal and movement barriers. Glob. Chang. Biol. **23**, 4970–4986 (2017). 28500795 10.1111/gcb.13760

[30] L. Comte, J. D. Olden, Climatic vulnerability of the world’s freshwater and marine fishes. Nat. Clim. Chang. **7**, 718–722 (2017).

[31] L. Comte, J. Murienne, G. Grenouillet, Species traits and phylogenetic conservatism of climate-induced range shifts in stream fishes. Nat. Commun. **5**, 5053 (2014). 25248802 10.1038/ncomms6053 PMC5898465

[32] T. P. Dawson, S. T. Jackson, J. I. House, I. C. Prentice, G. M. Mace, Beyond predictions: Biodiversity conservation in a changing climate. Science **332**, 53–58 (2011). 21454781 10.1126/science.1200303

[33] C. Parmesan, C. Duarte, E. Poloczanska, A. J. Richardson, M. C. Singer, Overstretching attribution. Nat. Clim. Chang. **1**, 2–4 (2011).

[34] R. A. Hastings , Climate change drives poleward increases and equatorward declines in marine species. Curr. Biol. **30**, 1572–1577 (2020). 32220327 10.1016/j.cub.2020.02.043

[35] L. A. Rutterford, S. D. Simpson, B. Bogstad, J. A. Devine, M. J. Genner, Sea temperature is the primary driver of recent and predicted fish community structure across Northeast Atlantic shelf seas. Glob. Chang. Biol. **29**, 2510–2521 (2023). 36896634 10.1111/gcb.16633

[36] W. W. L. Cheung, R. Watson, D. Pauly, Signature of ocean warming in global fisheries catch. Nature **497**, 365–368 (2013). 23676754 10.1038/nature12156

[37] K. Chen, J. D. Olden, Threshold responses of riverine fish communities to land use conversion across regions of the world. Glob. Chang. Biol. **26**, 4952–4965 (2020). 32564461 10.1111/gcb.15251

[38] C. Pringle, What is hydrologic connectivity and why is it ecologically important? Hydrol. Proc. **17**, 2685–2689 (2003).

[39] L. Comte, J. D. Olden, P. A. Tedesco, A. Ruhi, X. Giam, Climate and land-use changes interact to drive long-term reorganization of riverine fish communities globally. Proc. Natl. Acad. Sci. U.S.A. **118**, e2011639118 (2021). 34155095 10.1073/pnas.2011639118 PMC8271677

[40] J. A. Babaluk, J. D. Reist, J. D. Johnson, L. Johnson, First records of sockeye (*Oncorhynchus nerka*) and pink salmon (*O. gorbuscha*) from Banks Island and other records of Pacific Salmon in Northwest Territories, Canada. Arctic **53**, 161–164 (2000).

[41] A. Almodóvar, G. G. Nicola, D. Ayllón, B. Elvira, Global warming threatens the persistence of Mediterranean brown trout. Glob. Chang. Biol. **18**, 1549–1560 (2012).

[42] K. M. Alofs, D. A. Jackson, N. P. Lester, Ontario freshwater fishes demonstrate differing range-boundary shifts in a warming climate. Divers. Distrib. **20**, 123–136 (2014).

[43] L. Comte, G. Grenouillet, Do stream fish track climate change? Assessing distribution shifts in recent decades. Ecography **36**, 1236–1246 (2013).

[44] R. Hickling, D. B. Roy, J. K. Hill, R. Fox, C. D. Thomas, The distributions of a wide range of taxonomic groups are expanding polewards. Glob. Chang. Biol. **12**, 450–455 (2006).

[45] D. J. Booth , Detecting range shifts among Australian fishes in response to climate change. Mar. Freshwater Res. **62**, 1027–1042 (2011).

[46] L. Comte , RivFishTIME: A global database of fish time-series to study global change ecology in riverine systems. Glob. Ecol. Biogeogr. **30**, 38–50 (2021).

[47] GBIF.org, Data from “GBIF occurrence download (2024)”. Figshare. 10.15468/dl.c97hn3. Accessed 3 December 2024.

[48] W. Zhi, C. Klingler, J. Liu, L. Li, Widespread deoxygenation in warming rivers. Nat. Clim. Change **13**, 1105–1113 (2023).

[49] M. F. Johnson , Rising water temperature in rivers: Ecological impacts and future resilience. WIREs Water **11**, e1724 (2024).

[50] H. O. Pörtner, R. Knust, Climate change affects marine fishes through the oxygen limitation of thermal tolerance. Science **315**, 95–97 (2007). 17204649 10.1126/science.1135471

[51] D. J. Isaak, R. E. Rieman, Stream isotherm shifts from climate change and implications for distributions of ectothermic organisms. Glob. Chang. Biol. **19**, 742–751 (2013). 23504832 10.1111/gcb.12073

[52] C. Román-Palacios, J. J. Wiens, Recent responses to climate change reveal the drivers of species extinction and survival. Proc. Natl. Acad. Sci. U.S.A. **117**, 4211–4217 (2020). 32041877 10.1073/pnas.1913007117 PMC7049143

[53] P. Szekeres , On the neglected cold side of climate change and what it means to fish. Clim. Res. **69**, 239–245 (2016).

[54] A. J. Davis, L. S. Jenkinson, J. H. Lawton, B. Shorrocks, S. Wood, Making mistakes when predicting shifts in species range in response to global warming. Nature **391**, 783–786 (1998). 9486646 10.1038/35842

[55] J. D. Olden , Conservation biogeography of freshwater fishes: recent progress and future challenges. Divers. Distrib. **16**, 496–513 (2010).

[56] D. Tickner, , Bending the curve on freshwater biodiversity loss: An emergency recovery plan. BioScience **70**, 330–342 (2020). 32284631 10.1093/biosci/biaa002 PMC7138689

[57] J. Heino, J. Erkinaro, A. Huusko, M. Luoto, “Climate change effects on freshwater fishes, conservation and management” in Conservation of Freshwater Fishes, G. Closs, M. Krkosek, J. Olden, Eds. (Cambridge University Press, 2015), pp. 76–106.

[58] K. T. Tuff, T. Tuff, K. F. Davies, A framework for integrating thermal biology into fragmentation research. Ecol. Lett. **19**, 361–374 (2016). 26892491 10.1111/ele.12579 PMC4794773

[59] K. D. Fausch, C. E. Torgersen, C. V. Baxter, H. W. Li, Landscapes to riverscapes: Bridging the gap between research and conservation of stream fishes. BioScience **52**, 483–498 (2002).

[60] C. Wolter, R. Arlinghaus, Navigation impacts on freshwater fish assemblages: The ecological relevance of swimming performance. Rev. Fish Biol. Fish. **13**, 63–89 (2003).

[61] D. G. Jenkins, Does size matter for dispersal distance? Glob. Ecol. Biogeog. **16**, 415–425 (2007).

[62] B. B. Chapman, C. Brönmark, J. Å. Nilsson, L. A. Hansson, The ecology and evolution of partial migration. Oikos **120**, 1764–1775 (2011).

[63] A. E. Cahill , How does climate change cause extinction? Proc. R. Soc. B: Biol. Sci. **280**, 20121890 (2013). 10.1098/rspb.2012.1890 PMC3574421 23075836

[64] A. L. Angert , Do species’ traits predict recent shifts at expanding range edges? Ecol. Lett. **14**, 677–689 (2011). 21535340 10.1111/j.1461-0248.2011.01620.x

[65] B. L. Brown, C. M. Swan, Dendritic network structure constrains metacommunity properties in riverine ecosystems. J. Anim. Ecol. **79**, 571–580 (2010). 20180874 10.1111/j.1365-2656.2010.01668.x

[66] M. Pacifici , Assessing species vulnerability to climate change. Nat. Clim. Chang. **5**, 215–224 (2015).

[67] S. M. Prober , Shifting the conservation paradigm: A synthesis of options for renovating nature under climate change. Ecol. Monog. **89**, e01333 (2019).

[68] J. T. Abatzoglou, S. Z. Dobrowski, S. A. Parks, K. C. Hegewisch, TerraClimate, a high-resolution global dataset of monthly climate and climatic water balance from 1958–2015. Sci. Data **5**, 170191 (2018). 29313841 10.1038/sdata.2017.191 PMC5759372

[69] M. Johnson climateR R package version 0.3.5. (2024) https://github.com/mikejohnson51/climateR

[70] O. Mohseni, H. G. Stefan, T. R. Erickson, A nonlinear regression model for weekly stream temperatures. Water Resour. Res. **34**, 2685–2692 (1998).

[71] M. Punzet , A global approach to assess the potential impact of climate change on stream water temperatures and related in-stream first-order decay rates. J. Hydrometeorol. **13**, 1052–1065 (2012).

[72] S. Chamberlain rgbif: Interface to the Global Biodiversity Information Facility API, R package version 3.7.9. (2024). https://CRAN.R-project.org/package=rgbif.

[73] J. W. Hollister, Elevatr: Access Elevation Data from Various APIs. R Package Version 0.99.0. (2023) https://CRAN.R-project.org/package=elevatr.

[74] R. Froese, D. Pauly, FishBase. World Wide Web electronic publication. www.fishbase.org. (Accessed 1 May 2024).

[75] C. Boettiger, D. T. Lang, P. C. Wainwright, rfishbase: Exploring, manipulating and visualizing FishBase data from R. J. Fish Biol. **81**, 2030–2039 (2012). 23130696 10.1111/j.1095-8649.2012.03464.x

[76] D. Bates, M. Maechler, B. Bolker, S. Walker, Fitting linear mixed-effects models using lme4. J. Stat. Softw. **67**, 1–48 (2015).

[77] J. Fox, S. Weisberg An R Companion to Applied Regression, 3rd Edition (Thousand Oaks, 2019).

[78] J. Fox, S. Weisberg, Visualizing fit and lack of fit in complex regression models with predictor effect plots and partial residuals. J. Stat. Softw. **87**, 1–27 (2018).

[79] C. D. Eager, standardize: Tools for standardizing variables for regression in R R package version 0.2.1 (2017). https://CRAN.R-project.org/package=standardize.

[80] D. Lüdecke, sjPlot: Data visualization for statistics in social science (R package version 2.8.15 (2023). https://CRAN.R-project.org/package=sjPlot.

[81] R Core Team, R: A Language and Environment for Statistical Computing (R Foundation for Statistical Computing, Vienna, 2024), https://www.R-project.org/.

[82] H. Wickham, ggplot2: Elegant Graphics for Data Analysis (Springer-Verlag, New York, 2016).

[83] A. Kuznetsova, P. B. Brockhoff, R. H. B. Christensen, lmertest package: Tests in linear mixed effects models. J. Stat. Softw. **82**, 1–26 (2017).

