# Supplementary material for: Climate warming drives population trajectories of freshwater fish
Source: Proc Natl Acad Sci U S A. 2024 Dec 9;121(51):e2410355121. doi: 10.1073/pnas.2410355121 (PMC11665863; doi:10.1073/pnas.2410355121)
Supplement: Supplementary file 1 — Appendix 01 (PDF) [file pnas.2410355121.sapp.pdf]

Climate warming drives population trajectories of freshwater fish

Timothy M. Brown, Joseph O'Connor, Martin J. Genner

Appendix

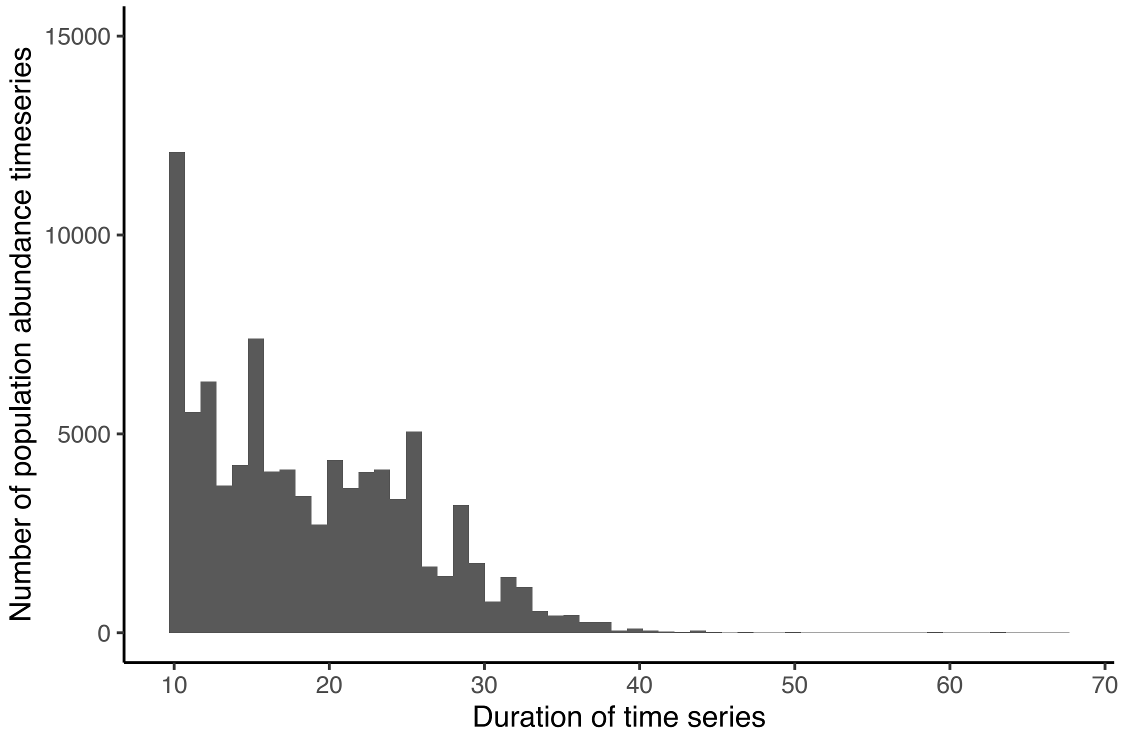

Figure S1 – Number of time series of each duration. All time series used had a minimum duration of 10 years. The maximum duration was 57 years.
